# Occupational Groups and Environmental Justice: A Case Study in the Bronx, New York

**DOI:** 10.5888/pcd15.180344

**Published:** 2018-11-15

**Authors:** Andrew R. Maroko, Brian T. Pavilonis

**Affiliations:** 1 The CUNY Graduate School of Public Health and Health Policy, New York, New York

## Abstract

We used spatial analyses to examine exposure of people in vulnerable occupational groups to neighborhood-level environmental pollutants in the Bronx borough of New York City. Five-year estimates of environmental ambient exposures (derived from land use regression models for PM2.5 [particulate matter with an aerodynamic diameter ≤2.5 µm] and black carbon) and demographic and occupational variables were harmonized at the census tract level. Correlations revealed that areas with high environmental exposures also had high proportions of people in service industries and manufacturing and high proportions of socioeconomically vulnerable populations. This combination of vulnerabilities may be cumulative, suggesting residents could have high occupational and residential exposures in addition to sociodemographic-related inequity.

## Objective

Socioeconomically disadvantaged populations and racial/ethnic minority populations often live in areas with more environmental hazards than other population groups, an environmental justice issue that may lead to poor health outcomes and worsen differences in health (1–4). However, few studies have examined how occupational groups may be differentially distributed with respect to ambient environmental (neighborhood) exposures. Our ecological study sought to determine whether people in vulnerable occupational groups (ie, those with potentially high exposures to pollutants in the workplace) could be overexposed to environmental pollutants on the basis of their place of residence in the Bronx borough of New York City, thus constituting a potential environmental justice issue.

## Methods

Employment information for civilians aged 16 or older at the census tract level were obtained from the US Census Bureau’s 2011–2015 *American Community Survey* via the National Historical Geographic Information System (NHGIS.org, IPUMS.org) (5). We collapsed job classifications into 4 categories on the basis of a previous study (6): white collar, service industry, construction (including protective services and agriculture because of a small sample size and similarity in exposure), and manufacturing.

Environmental exposures were derived from 300-meter resolution land use regression model outputs provided by the New York City Department of Health and Mental Hygiene (7). Land use regression uses a statistical model to estimate ambient pollutant concentration as a function of land use (eg, vehicle traffic, building emissions, population density). The environment surrounding monitoring locations in New York City was used to parameterize the regression equation for each year (number of monitors is from 60 to 100, depending on year), which is then applied to locations around the city where no measurements have been taken to create a continuous surface of annual average concentration estimates (8). We resampled land use regression outputs in the Bronx at the census tract level and calculated 5-year (2011–2015) average concentrations of PM2.5 (particulate matter with an aerodynamic diameter ≤2.5 µm) and black carbon (a type of particulate pollution often used as a marker for diesel exhaust [9]). Census tract-level demographic and socioeconomic variables (proportion of non-Hispanic white, non-Hispanic black, and Hispanic populations and the population’s poverty status) were derived from the *American Community Survey* 5-year data for 2011–2015 (5) (Figure). Associations among pollutant concentration, occupational groups, demographics, and economics were tested by using nonparametric Spearman correlations for census tracts with more than 200 residents (n = 330).

**Figure:**
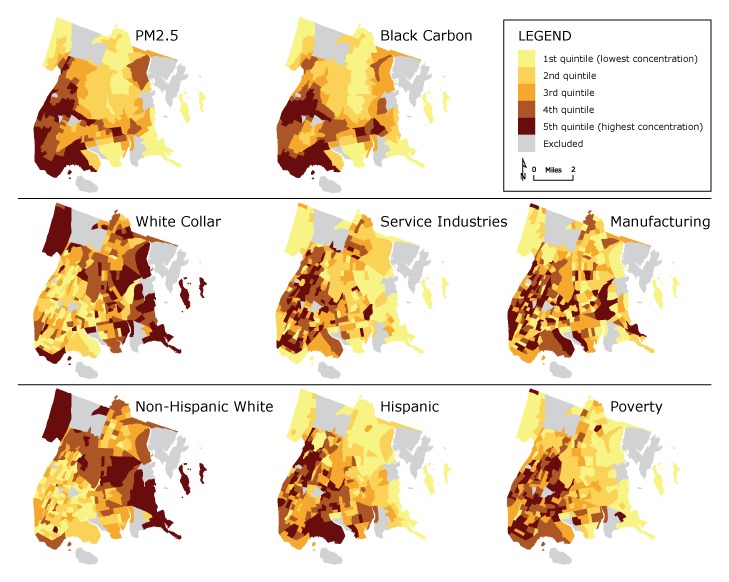
Spatial distribution of pollutants, occupational groups, and demographics, in quintiles by census tract, Bronx, New York, 2011–2015. The 2 pollutants are PM2.5 (particulate matter with an aerodynamic diameter ≤2.5 µm) and black carbon (a type of particulate pollution often used as a marker for diesel exhaust [9]). The occupational groups are white collar, service industries, and manufacturing (construction not shown but available from authors on request); the demographic groups are non-Hispanic white and Hispanic populations (non-Hispanic black not shown but available from authors on request) and poverty (people living below federal poverty guidelines). Tracts with low populations (200 or fewer residents) were excluded. Sources: *American Community Survey 2011–2015* ACS 5-Year Estimates via the National Historic Geographic Information System (5), New York City Community Air Survey 2011–2015 (10).

## Results

Spearman correlations identified significant positive associations between estimated concentrations of black carbon and PM2.5 and proportions of Hispanic residents and people with incomes below federal poverty guidelines (*P* < .01). The proportion of non-Hispanic black residents was not significantly associated with estimated pollutant exposures. Significant positive associations (*P* < .01) were observed between census tracts with high proportions of white-collar workers and non-Hispanic white residents. Conversely, negative associations were found between the proportion of white-collar workers and the proportion of non-Hispanic black and Hispanic residents and people living in poverty (*P* < .01). Census tracts with high proportions of service industry or manufacturing workers were negatively associated with non-Hispanic white populations but positively associated with Hispanic populations and with people living in poverty (*P* < .01). Proportions of non-Hispanic black residents were positively associated with service industry occupations (*P* < .01) but did not reach significance with respect to manufacturing.

The proportion of workers who identified as being employed in the service industry or manufacturing had significant positive associations with ambient environmental exposure to black carbon and PM2.5 (*P* < .01). Conversely, tracts with high proportions of white-collar workers had significant negative associations with these pollutants (*P* < .01) (Table).

**Table:** Spearman Correlations for Occupational Groups, Demographics, and Environmental Exposures, Bronx, New York, 2011–2015

Variables^a^	White Collar	Service Industry	Manufacturing	Construction^b^	Non-Hispanic White	Non-Hispanic Black	Hispanic	Poverty^c^	PM2.5^d^	Black Carbon
**Occupation**										
White collar	1	^—e^	^—e^	^—e^	^—e^	^—e^	^—e^	^—e^	^—e^	^—e^
Service industry	−.868^f^	1	^—e^	^—e^	^—e^	^—e^	^—e^	^—e^	^—e^	^—e^
Manufacturing	−.554^f^	.310^f^	1	^—e^	^—e^	^—e^	^—e^	^—e^	^—e^	^—e^
Construction	−.219^f^	−.089	−.041	1	^—e ^	^—e^	^—e^	^—e^	^—e^	^—e^
**Demographics**										
Non-Hispanic white	.594^f^	−.605^f^	−.307^f^	−.017	1	^—e^	^—e^	^—e^	^—e^	^—e^
Non-Hispanic black	−.217^f^	.242^f^	.032	.074	−.507^f^	1	^—e^	^—e^	^—e^	^—e^
Hispanic	−.598^f^	.564^f^	.446^f^	−.011	−.516^f^	−.222^f^	1	^—e^	^—e^	^—e^
Poverty	−.676^f^	.682^f^	.394^f^	−.025	−.619^f^	.167^f^	.648^f^	1	^—e^	^—e^
**Environmental pollutant**										
PM2.5	−.503^f^	.529^f^	.349^f^	−.122^g^	−.495^f^	.010	.636^f^	.579^f^	1	^—e ^
Black carbon	−.505^f^	.511^f^	.356^f^	−.071	−.500^f^	−.024	.638^f^	.584^f^	.949^f^	1
**Mean (standard deviation)**	48.51 (12.53)	29.39 (9.75)	11.13 (4.46)	10.97 (4.48)	12.84 (20.74)	28.76 (20.53)	52.67 (20.88)	29.78 (15.03)	9.71 (0.60)	1.19 (0.16)

a Occupational and demographic values are percentage of the census tract population. Environmental pollutant values are concentrations at the census tract level.

b Includes protective services and agriculture.

c Percentage of the census tract population with incomes below federal poverty guidelines.

d Particulate matter with an aerodynamic diameter ≤2.5 µm.

e The correlation matrix is symmetrical. Entries to the right are suppressed for clarity.

f Correlation is significant at *P* < .01 level (2-tailed).

g Correlation is significant at *P* < .05 level (2-tailed).

## Discussion

The Bronx borough of New York City has often been studied with respect to environmental justice issues because of its high proportion of vulnerable populations, historic settlement patterns, environmental burdens, and poor health outcomes among its residents (11,12). However, occupational exposures to airborne particulate matter are often overlooked in the development of chronic diseases such as cardiovascular disease and, depending on the industry, can be orders of magnitude larger than environmental exposures (13). Occupational sectors such as service industry, construction, and manufacturing have higher mortality rates than white-collar sectors (6).

The results from our study show several spatial relationships among occupational groups, neighborhood environmental exposures, and demographics. The most vulnerable occupational groups (ie, those with the highest likelihood of poor health outcomes or high exposure to pollutants in workplace environments) are positively associated with neighborhoods with higher concentrations of PM2.5 and black carbon. These same neighborhoods also tend to have higher proportions of vulnerable populations on the basis of race/ethnicity and income levels. These sociodemographic characteristics are associated with increased risk of environmental exposures and possibly amplify the effects of these exposures (4). These populations are consistently associated with increased incidence and severity of disease — potentially as a function of psychosocial stressors such as discrimination and social exclusion (4). This combination of vulnerabilities is likely to be cumulative, putting residents of certain neighborhoods in double jeopardy on the basis of traditionally measured environmental injustices as well as environmental injustice as a function of occupational group. Such residents could have high exposures both at work and at home and may suffer from additional socially driven inequity based on racial/ethnic or economic characteristics.

Occupational attributes appear to be important variables, not only with respect to environmental justice work but also more generally in terms of environmental health studies. Although such studies often incorporate either neighborhood exposures or occupational exposures, they rarely include both simultaneously. The confluence of high-risk occupational groups and environmental neighborhood exposures (physical and social) may further contribute to, or exacerbate, health disparities in regions like the Bronx.
